# Artistic Anatomy and the Sciences Useful to the Artist

**Published:** 1883-02

**Authors:** S. V. Clevenger

**Affiliations:** Professor of Artistic Anatomy, Art Institute, Chicago


CHICAGO MEDICAL

JOURNAL & EXAMINER.

Vol. XLVI.—FEBRUARY, 1883.—No. 2.

### Original Communications.

### Article I.

Artistic Anatomy and the Sciences Useful to the Artist. Opening Lecture of a Course Delivered at the Winter Term, 1883, by S. V. Clevenger, a.m., m.d., Professor of Artistic Anatomy, Art Institute, Chicago.

One of the best things Sir Joshua Reynolds ever said was: "A painter stands in need of more knowledge than is to be picked off his palette, or collected by looking on his model, whether it be in life or in picture. He can never be a great artist who is grossly illiterate." * After mentioning the advisability of being conversant with the poets, "that he may imbibe a poetical spirit," the recommendation is made to study that "philosophy which gives an insight into human nature, and relates to the manners, character, passions and affections." "The

* Reynolds' Seventh Discourse Delivered to the Students of the Royal Academy, December 10, 1776.

artist," said Sir Joshua, "ought to know something concerning the mind, as well as a great deal concerning the body of man." Admitting all this as self-evident and trite, it is to be observed, that in the days of these utterances, knowledge was not so readily attainable as now, and, with the exception of gross anatomy, which at that time had been thoroughly worked out, there was as much of the false as of the true in the sciences available to the artist; mental science especially.

It is not to be expected that intimacy with the sciences will create an artist, for the artist, like the poet, is *nascitur*, *non fit*; but certainly the born artist will be all the greater and better for acquired knowledge, as will be the born poet for the mastery of prosody.

If we are to believe Haydon, Sir Joshua was but an indifferent chemist, and knew but little of anatomy. At once the uninformed painter would ask, Why should the artist be familiar with chemistry? All your colors are chemical combinations, which in mixing undergo certain color reactions or changes which are not permanent in all cases; some pigments fade; others darken, and still others pass through a change or many changes. A superficial knowledge of chemistry will enable the modern painter to avoid the errors of the old masters, whose paintings, for the most part, are not now as originally created. I notice a disposition which is to be encouraged, on the part of recent art authors,* to set forth in detail the physics involved in color blending.† While for mere copying or picture-making, a little knowledge of chemistry and physics may answer, in this day, if the painter aspire to represent a modicum of what his art is capable of setting forth by the aid of modern science, were he to attempt a knowledge of mental science, to know the mysteries of the emotions, "manners, characters, passions and affections," nothing short of a *good* acquaintanceship with physics, chemistry, biology, physiology and anatomy would suffice.

This lecture is intended as the first of a series upon artistic anatomy. We will find our subject comprehensive enough not to admit of digression.

---

* Blanc, Grammar of Art—Translation by Kate N. Doggett.

† An excellent work for the artist to read on this subject is Von Bezold's Theory of Color.

Galen describes the human bones accurately enough, but it appears that very little was accomplished by the ancient Greeks in anatomical studies until the days of Erasistratus, who enjoyed the rare privilege of dissecting criminals alive. Flaxman, * whose lectures on sculpture I advise every artist to read, gives in detail the evolution of art anatomy so far as it is known. John Marshall † prefaces his bulky volume entitled Anatomy for Artists, with a very interesting history of this subject. The remainder of Marshall's work will answer very well for reference. Fau ‡ affords us, in cheap, condensed form, some very beautifully executed illustrations of the human anatomy, but his text is worthless. Haydon,|| old as his work is, in a few pages of his second and third lectures, gives the most concise, and, to the artist, valuable description of the bones and muscles extant. Fau's plates, Haydon's lectures, with a skeleton and cadaver, will answer, though there are some essential things omitted by Haydon and matters introduced, which are unimportant. Hodges' little book § added, will leave nothing to be desired, unless the artist wish to excel in anatomy, in which case he can have recourse to the larger medical works.¶ But there is danger of Haydon's fate being encountered, in mistaking or substituting the means for the end. The painter or sculptor may cease to be an artist in too toilsome anatomical work.

In the many positions possible for the body to assume, there are exhibited certain surfaces, protuberances, ridges, depressions, prominences, curves, angles and lines produced by parts of bones, joints, muscles, tendons and veins, partially obscured by layers of fat, all beneath an investing sheet of cuticle. To be able to depict the outer form properly, the artist must know something of the underlying tissues, just as the sculptor seeks first the exact contour of body and limbs, before clothing them with clinging drapery.

---

* Flaxman, Lectures on Sculpture.

† John Marshall, Anatomy for Artists.

‡ J. Fau, Elementary Artistic Anatomy. Translated by Blake.

|| B. R. Haydon, Lectures on Painting and Design, Vol. I.

§ Hodge, Practical Dissections.

¶ Henle, Sharpey and Quain, Gray, Leidy, Holden, H. Allen, etc.

It is probable that the early Greek artists contented themselves with externals, to which fact many defects are attributable. According to Vitruvius, geometrical rules were applied by them, however, two of which we have in the human figure included in a circle and square, the fingers of the outstretched hands and arms touching the periphery.

The male body was also divided by the Greeks into eight heads. Fau modifies from Jean Cousin's * measurements as follows: Beginning at the summit of the head, and ending at the heel, the eight heads are included by lines drawn through the lower part of the chin, nipples, navel, pubis, middle of thigh, knee, below the calf. The head is divided into four noses, similarly, at hair line, origin of nose, upper lip, end of chin; a nose length lies between chin and supra-sternal fossa. The other proportions, as well as those mentioned, are best represented by diagrams at hand in the Academy. For details I refer you to Flaxman, Fau, and future lectures.

With anatomy as an indispensable groundwork, the artist soon finds himself erecting a superstructure of physiology, physiognomy, and thereupon analyses of beauty and the emotions.

The contraction of the muscles of the face in differing degrees and combinations, have come to be recognized as exhibiting certain inward conditions, called emotions, mainly those of pleasure or displeasure; a pleasing symmetry of form and benignity of expression is styled beauty; and from time out of mind, physiognomists have sought to read the secrets of others in the appearance of the face, and in the demeanor. The nervous action which results in expression; the contraction of muscles; the distension of the veins; all these, and more such which the artist must consider, are physiological processes.

Beauty of form is a strictly anatomical subject. To the thoughtless, it is an indefinable appearance which pleases; to the philosopher it is much more. Its appreciation is governed by the law of relativity, as is everything else in the universe; and every nation, nay, every individual, has its and his own standard. In portraying the queen of heaven, the Madonna was Italian, French,

---

* *L'art de desseigner de maistre* Jean Cousin, Paris, 1680.

English, Spanish, Flemish or Dutch, according to the nationality of the artist. The Hollander favored a rotundity of form which others would call fat and squab. The African, Asiatic, American and European types differ immensely, indeed, to a provincial extent, among themselves; so much so that beauty on one side of a river, mountain range or political boundary, would appear hideousness to the inhabitants of the other side. Symmetrical softness of outline, in conjunction with nicely blended colors, seems to be, *nearly*, the universal ideal of the graceful and beautiful. Hogarth * attempted the general application of his wave line, not only to beauty of animal form, but to architecture, landscape, etc. He carried his single principle too far, for Gothic architecture is attractive in its angularity, and a rugged landscape may be beautiful without the presence of a curve. In general terms, however, Hogarth was right. Edgar Poe's words here seem appropriate: † "The mathematicians afford us no more absolute demonstrations, than the sentiment of his art yields to the artist. He not only believes, but positively knows, that such and such apparently arbitrary arrangements of matter, or form, constitute, and alone constitute, the true beauty. Yet his reasons have not yet been matured into expression. It remains for a more profound analysis than the world has yet seen, fully to investigate and express them." Poe said aright, that it was not allotted to the artist to analyze his instincts and sentiments. In another connection we will see that the artist himself must needs be analyzed first, and it was not till Herbert Spencer's day that this was done. To the artist beauty is a sentimental consideration; to Spencer sentiment is a matter susceptible of explanation. Hence the Spencerian analysis of beauty should be sought for by the artist.

In his Nov. 10, 1759, letter to the *Idler*,‡ Sir Joshua reviews this matter fairly, for the time in which he lived. He alludes to habit or custom making, in a certain sense, white black, and black white. He supposes that "were an Ethiopian to paint the

---

* The Analysis of Beauty, written with a view of fixing the fluctuating ideas of taste. By William Hogarth. London, 1810.

† The Landscape Gardener. Edgar A. Poe.

‡ Reynolds' Works, Vol. II, p. 134.

Goddess of Beauty, he would represent her black, with thick lips, flat nose, and woolly hair. We, indeed, say that the form and color of the European is preferable to that of the Ethiopian; but know of no other reason we have for it but that we are more accustomed to it." * * * * "From what has been said, it may be inferred that the works of Nature, if we compare one species with another, are all equally beautiful, and that preference is given from custom, or some association of ideas, and in creatures of the same species, beauty is the medium or center of all its various forms." Aristotle writes:* Every art, every method and every institution, every action and council, seems to seek some good; therefore, the ancients pronounced the beautiful to be the good." There was a time when it was flat heresy to dissent from Plato or Aristotle. We are to-day privileged in a measure to use our own brains, and may safely pronounce the assertion a sentimental one, which strikes the heart strings tunefully, but for all that, it is pure jingle, bad logic; conclusion absurd and *non sequitur.* Read Spencer's Data of Ethics,† if you wish to see the reasons for conceiving the beautiful and the good to be identical. In the shortest possible language, it is a praiseworthy delusion; it is because we want to do so, not because there is any inherent identity.

Alluding again to the fact that habit, custom or education governs our regard for beauty, Hogarth‡ calls attention to the exquisite sinuosities of the human bones. Hogarth had familiarized himself with the skeleton in seeking his lines of beauty therein, and speaks of the "beautiful ossa innominata," while young lady pupils are very apt to shudder when first shown a skeleton, and pronounce it disgusting and horrible. It is a very common thing for anatomists to go into as many ecstacies over an exhibition of beautifully proportioned bowels in the dead body as a lady would over a duck of a bonnet, yet ladies may become naturalists and discuss with their intellectual peers the cunningness and loveliness of snails, spiders, ear wigs and angleworms. Similarly we all laugh at the fashions of dress of a few years back

---

* Aristotle, Treatise on Morals.

† Herbert Spencer's Data of Ethics. Elements of Psychology.

‡ Analysis of Beauty, p. 55.

just as we will wonder a few years hence how we could have been so absurd as to dress as we do to-day.

Aside from Hogarth's attempt to make too much of the curve idea, his Analysis is well worth reading. He has collected therein many useful hints not to be found in other authors; he notes for instance: "There is another very extraordinary circumstance which nature hath given us to distinguish one age from another by, which is, that though every feature grows larger and larger, until the whole person has done growing, the sight of the eye still keeps its original size; I mean the pupil with its iris or ring; for the diameter of this circle continues still the same, and so becomes a fixed measure by which we, as it were, insensibly compare the daily perceived growings of the other parts of the face and thereby determine a young person's age. You may sometimes find this part of the eye in a new-born infant fully as large as in a man of six feet, nay, sometimes larger. In infancy the faces of boys and girls have no visible difference, but as they grow up the features of the boy get the start and grow faster in proportion to the ring of the eye than those of the girl, which shows the distinction of the sex in the face. Boys who have larger features than ordinary in proportion to the rings of their eyes, are what we call manly featured children as those who have the contrary, look more childish and younger than they really are. It is this proportion of the features with the eyes that makes women when they are dressed in men's clothes look so young and boyish; but as nature doth not always stick close to these particulars, we may be mistaken both in sexes and ages." The facts to which Hogarth calls attention may be summed up to the effect that no matter what changes growth may cause in the relative sizes of faces, at all ages and in both sexes the absolute size of the pupil remains the same. There is a physical explanation in the retina of all alike requiring the same amount of light for visual impressions. The adult can see no better or more than the infant, and this is often true in more senses than one.

An important item concerning the eye seems to be ignored by most writers upon art. The cornea or glassy part of the eye is a small globe segment laid upon the sclerotic or white of the eye, the eyeball having a larger radius than the cornea. This appears to have been known to many of the ancients though not faithfully reproduced in the casts taken from their statuary. Some sculptors even of acknowledged eminence have what appears to me to be the abominable trick of gouging a hole in the globe to give the effect of reflection. This might be justifiable in colossal figures but in fine statues it would be better art, certainly truer art, to carve the eye as it actually is, with its superimposed cornea.

You will encounter many pretentious works that profess to unravel the secrets of the human face. The versatile Rimmer * has contributed a ponderous set of drawings and "observations" to the physiognomical muddle. His analytical text and classifications are simply soul-harrowing in their artificiality. Sir Charles Bell † was the first to contribute to the subject scientifically, but the windy Zurich parson, Lavater,‡ is more widely known as a writer upon physiognomy because his vaporings suited the superficial readers who always outnumber the thinkers. Strip Lavater's books of their ejaculations, and they undergo great reduction in size. He seems to have been lost in rapturous admiration of his own conceits. He finally compresses his views into one hundred rules, which carefully read yield six per cent. of truth, twenty-four per cent. of downright malevolence, and seventy per cent. of nonsense. I am sure the ladies will not consider this attack a harsh one when they are told that Lavater's seventy-first rule begins: "Vanity or pride is the general character of all women."

The physiognomy of the past is the childish attempt to judge gentility by dress. A beautiful face is a good face; only the wicked dress shabbily, etc., although food for reflection was furnished by some facts like these: Socrates had a stupid, villainous looking head; Christ is recorded as possessing no comeliness, and St. Paul's appearance was insignificant. There is no denying the justness in many, possibly the majority of cases, of the childish estimate, which but slightly modified, is the same as our own off-hand determination of character by appearance, but if we ask

* Dr. William Rimmer, Art Anatomy, Boston, 1877.

† Sir Charles Bell, Anatomy and Philosophy of Expression, 1806.

‡ J. C. Lavater, Essays on Physiognomy.

ourselves *why* does such a face impress us as being a "good" one and another as being "bad," we must go deeper than the skin. Take the favorable instance of a really kind-hearted, upright person in fair circumstances and good health, with the purely accidental accompaniment of regular features and fine complexion. The goodness in him shows in his face, we say. Why? Because he is not distressing himself with unnecessary schemes and cares, and his consciousness of having harmed no one, nor wishing one harm, enables him to look you fairly in the eye, without the impudence of the domineering person, who can do the same only in a disagreeable way. Your good man is usually so self complacent and good humored that his eye twinkles and his face is wreathed in smiles, and that is about all there is in the so-called "good face." Cannot each one of us recall some such face with which we were prepossessed only to discover in time that we had been deceived in the possessor? To convince you how we often err in the opposite direction it is worth mentioning that defective eyes impart an appearance of character wholly undeserved by the individual estimated. Thus convergent strabismus and contracted pupils give sinister expressions to the face; divergent strabismus a foolish look; while dilated pupils or protuberant eyes express a surprise which the persons afflicted with them may be far from feeling. Lavater's method of discrimination was carried to an absurd degree by a wealthy St. Louis merchant during my boyhood. He declared that without exception, every one-eyed man was a scoundrel. Soon after making this brilliant remark he lost an eye himself, and to add to the whimsicality he happened to be a pretty bad fellow. The poetical justice of the accident impressed itself firmly on my mind.

Winckleman said that superstitious reverence for the works of their ancestors prevented improvement among the Egyptians. Apropos of this, with much less truth, Disraeli causes one of the characters in his last novel to remark, that there is nothing original about the Americans, their ideas, their institutions, their arts are all borrowed, even their language is imported. There is an undeniable toadyism among the snobs of America, but it may be truly said that we imported both toadyism and snobbery from our mother country. In the matter of art we affect a reverence for the old masters with all the insincerity of our English ancestors. Listen to these words of the first president of the Royal Academy: "The keeper of the Vatican told me that it has frequently happened that many of those whom he had conducted through the apartments have asked for the works of Raphael, and would not believe that they had already seen them, so little impression had these performances made on them. One of the first painters of France once told me that this circumstance happened to himself, though he now looks on Raphael with that veneration which he deserves from all painters and lovers of art. I remember very well my own disappointment when I first visited the Vatican; but in confessing my feelings to a brother student of whose ingenuousness I had a high opinion, he acknowledged that the works of Raphael had the same effect on him, or rather that they did not produce the effect which he expected. This was a great relief to my mind, and on inquiring further of other students, I found that those persons only who from natural imbecility appeared to be incapable of relishing those divine performances, made pretentions to instantaneous raptures on first beholding them."

We thus have authoritative sanction for the belief that genuine admiration for the ancient in art, simply because it is ancient, can only come by cultivation, just as we learn to relish classical music or Limburger cheese. Please understand that I am far from disparaging old artistic productions. There have been undeniably very many grand statues and pictures worked out by gifted brains and hands in the early centuries, but, by all that is honest, do let us use our own eyes and determine for ourselves the merits of art work. Did it ever occur to you that "recognition" can be largely bought and that many sublime modern works are this day feeding rats and moths and that their authors are starving in attics because the accident of "recognition" has not happened to them? What up-hill work Reynolds and Benjamin West would have experienced had they not met with Royal favor. The want of it killed poor Haydon, and Elizabeth Thompson's fortune was made when Victoria purchased her "Roll-Call," after the artist had been repeatedly "skied" by the Academy. One of Raphael's pictures, his "Madonna dei Candelabri," has arrived in New York, and all snobdom rushes to view and praise it. That same group of "spirituelle" heads and candles might have hung till doomsday in its present place without attracting more than casual notice, had its authorship been unannounced. It is my business as your scientific teacher to bid you open your eyes to the defects in your arts and artists. You can get all the sincere and insincere adulation you may desire for the objects of your reverence from many other sources. To quote from Sir Joshua again: "I consider you as arrived at that period when you have a right to think for yourselves and to presume that every man is fallible; to study the masters with a suspicion that great men are not always exempt from great faults; to criticise, compare and rank their work in your own estimation, as they approach to, or recede from, that standard of perfection which you have formed in your own minds, but which those masters themselves, it must be remembered, have taught you to make. It is their excellencies which have taught you their defects."

"Nor would we have it thought" says Hogarth, "that the ancients or moderns have yet come up to the utmost beauty of nature. Who but a bigot, even to the antiques, will say that he has not seen faces and necks, hands and arms in living women, that even the Grecian Venus doth but coarsely imitate?" It is said that Barnum had on exhibition the skull of Washington when he was a boy, but that is no worse than the anachronism of Rubens, who represented St. John the Baptist as as elderly man while Christ was a child, in the same picture. An altar-piece presumably of the Flemish school, depicts Abraham about to sacrifice Isaac with a blunderbuss. This seems absurd enough, but what must we think of deliberate anachronisms such as the Hyde Park Lord Wellington apeing Achilles, and the Greenough Washington as Jupiter Olympus with tunic and toga. There is one, among several, good features in this statue; it was too large to be placed in the rotunda so it now sits looking toward the senate wing of the Capitol, while the right hand points toward the old Capitol prison.

Samuel Kneeland* deprecates the indulgence by the artist in

* The Monstrous in Art, *Popular Science Monthly*, p, 731, vol, xiv.

worn out mythological conceptions. He dubs the centaurs and sundry other hexapod mammal impossibilities as monstrous, and concludes: "Fidelity to truth cannot injure anything but the false and the temporary. The essentially beautiful must be in nature; it cannot be beyond it, above it, nor below it; the merely ideal in form can have no real existence in the mind of man."

Falsification has had many powerful upholders. The first lecturer at the Royal Academy decried fidelity in historical pictures. Generals must not be represented one-armed, one-eyed, or otherwise maimed if they really were so; the likeness must be sacrificed to the conception of heroic form. He criticised Bernini's David as undignified because the figure bit the nether lip while using the sling—a most natural attitude. Our Phil. Sheridan would have been painted by this "nature mender" as a giant, and Abraham Lincoln as an Adonis. As an outcome of that sentiment we have De Soto just out of a bandbox on the banks of the Mississippi, and Washington about to make New Year's calls across the Delaware—judging from his apparel.

You will, during the remainder of your lives, meet with enough blind adoration of the old masters and their executions to enable you to stand a little fault-finding. It requires some time for an anatomist to recover from such a shock as this: There are groups of muscles on the forearm known as flexors, because they flex or bend the wrist toward the shoulder. It is a matter of very easy demonstration, that the group called flexor digitorum sublimis has its origin at the inner condyle of the humerus, the inner side of the coronoid process of the ulna and oblique line of the radius. It would be thus well thrown at its origin toward the ulnar or inner side of the arm. In Rembrandt's "Dissecting Room" or "Anatomy at the Hague," 1632, Prof. Nicholas Van Tulp appears to be demonstrating to the guild of Amsterdam surgeons, an anomaly not on record. As well as can be made out, the picture shows this muscle coming from the radial or outer side, the outer condyle of the humerus.

Reverting to the intentional false in art, and to the report that there is not a reliable Grecian likeness extant, Tuckerman *

* H. T. Tuckerman's Book of Artists, p, 605.

describes two busts of Daniel Webster, by Hiram Powers and another American sculptor. Powers idealized Webster's head into that of a demi-god. The other artist made a faithful likeness. In Tuckerman's words "mathematically correct in dimensions and feature, wholly unidealized, and therefore universally recognized and prized by personal acquaintances of the statesman; in the hotels he frequented, in the homes of his friends, and in public halls, this bust is constantly seen." The friends of Webster favored the accurate likeness, but as they passed away the fanciful bust of Powers contended for a share of popular favor. At last the succeeding generations came to know the truth and even official recognition (questionable but significant honor) was not awarded Power's *Jovian* Webster, but the fifteen cent postage stamps are adorned with an engraving from the bust of *Daniel* Webster.

If a likeness is not a likeness, should it usurp the place of one? A deserved fate awaits the falsification of the historian, the artist, and any one who hopes to have his works outlive him, and who tries to perpetuate a lie in ink, in colors or in stone. Imagine the archæologist unearthing Greenough's Washington, or the Hyde Park Wellington. The attire of those figures would be to him an unwarrantable anachronism, not to be explained away. Admit Reynolds' historical falsehood into your pictures, and have future ages say of you, "Nothing reliable in their art; no dependence upon their figures as representations." Everybody modern is somebody else ancient.

Let us now look at a more pleasing and hopeful aspect of art, which walks hand in hand with science. As science ever instructs and assists art, so does art assist, elevate and perpetuate science. They are inseparable companions, coming to each other's rescue, when, as they journey, one or the other lags by the way. Up to the beginning of this century, the arts developed much more rapidly than the sciences, with the exception of the mathematical. There is a reason for it in the evolution of society. The ornamental precedes the useful, among all races; similarly, the emotional and sentimental is developed before the reasoning faculties, and furthermore, the emotions have suppressed, and always will, to a greater or less degree, trample upon and hinder the progress of the intellectual.

I appeal to the history of the world for verification. The beginning of history shows man to have been a true savage everywhere, with the emotionalism and want of reflection of a child. As he passed into barbarism, he merely refined his savage desire for display. He was but little less bloodthirsty; he grew more artistic in his decorative ability, but cared little for science, except as it aided his brawls and crusades. The barbarian merges by slow degrees into civilized man, and we behold sculpture, painting, and decorative arts greatly developed, while John Calvin burns the anatomist Servetus alive, while Giordano Bruno is destroyed at the stake, Galileo is imprisoned, Copernicus anathematized, Roger Bacon's chemical revelations rewarded by the monks 'dungeon. But, we say, a better time has come, in which the scientist may speak and write as he wishes. True, we no longer burn and hang for opinions' sake; we have so refined our cruelty that we do even worse, we ostracize, ignore and sneer at living truths, and the men whose shoes we are not worthy to blacken, conscientious men, who have devoted their lives and rare intellects to the elucidation of problems of the utmost importance to their fellow-men. Science has taken a stride in the last twenty years, through the exertions of two men in England, the importance of which art is slow to comprehend. There has been recently laid at rest in Westminster Abbey, by the side of Sir Isaac Newton's remains, the body of Charles Darwin, who during his lifetime was made the butt of the brainless mob. It is only within a few years that the sapient editorial and ecclesiastical mind has gained a modicum of appreciation of the genuineness of his labor, and it is as amusing as it is disgusting to know that one of our city pulpit orators, the celebrated "assimilator," who had for years been damning Darwin and his works, amazed at the unimpeachable testimony brought forward to show the purity and sincerity of the philosopher's life, alluded to him, when dead, as the *saintly* Darwin.

Charles Darwin's "Expression of the Emotions in Man and Animals;" is a book every artist should own and study. It sets forth, in the most convincing manner, the reasons for every contortion of face and body which the artist can depict. I will attempt to pass rapidly in review a few of the deductions, referring you to the book for the methods by which these were attained. Three main principles underly expression :

I. The principle of serviceable associated habit.

II. The principle of antithesis.

III. The principle of action due to the constitution of the nervous system, independently from the first of the will, and independently, to a certain extent, of habit.

Each principle could afford us subject-matter for a series of lectures, so we must be content with a few illustrations. A person threatened with some imminent danger, as a blow, or something falling upon him, involuntarily jumps away, closes his eyes, and stretches out his hands to ward off the injury. Now, this is a serviceable action, evidently, but the same person may perform the same maneuvers upon hearing some startling, unpleasant intelligence, without immediate danger, or any at all, to the listener; for instance, the announcement of the death of a friend.

"Many carnivorous animals, as they crawl toward their prey, and prepare to rush or spring on it, lower their heads and crouch, partly, as it would appear, to hide themselves, and partly to get ready for their rush ; and this habit, in an exaggerated form, has become hereditary in our pointers and setters. Now I have noticed, scores of times, that when two strange dogs meet on an open road the one which first sees the other, though at the distance of one or two hundred yards, after the first glance always lowers its head, generally crouches a little, or even lies down; that is, he takes the proper attitude for making a rush or spring, although the road is quite open and the distance great. Again, dogs of all kinds, when intently watching and slowly approaching their prey, frequently keep one of their fore-legs doubled up for a long time, ready for the next cautious step, and this is eminently characteristic of the pointer. But from habit they behave in the same manner whenever their attention is aroused. I have seen a dog at the foot of a high wall, listening attentively to a sound on the opposite side, with one leg doubled up; and in this case there could have been no intention of making a cautious approach." This sufficiently illustrates the first principle, though it embraces an immense range of expression.

Next, "When a dog approaches a strange dog or man in a savage or hostile frame of mind, he walks upright and very stiffly, head raised, tail erect and rigid, hairs bristling, pricked ears directed forward, eyes staring fixedly. These actions follow from the dog's intention to attack his enemy. As he prepares to spring with a savage growl on his enemy, the canine teeth are uncovered, and the ears pressed close backward on the head. Let us now suppose that the dog suddenly discovers that the man whom he is approaching is not a stranger, but his master; and let us observe how completely and instantaneously his whole bearing is reversed. Instead of walking upright, the body sinks downward, or even crouches, and is thrown into flexuous movements; his tail is lowered and wagged from side to side; his hair becomes smooth, ears depressed and drawn backward, but not closely to the head, and his lips hang loosely; the eyes no longer appear round and staring. These motions are explicable solely from being in complete opposition or antithesis to the attitude and movements, which, from intelligible causes, are assumed when a dog intends to fight, and which, consequently, are expressive of anger."

The cringing stoop of human humility is antithetical to the attitude of pride or defiance.

Trembling of the muscles is accounted for by the third principle—it is due to the escape of nerve force. Similarly laughter may, when not sufficing to relieve the emotion of merriment, be supplemented by a burst of tears, by way of escape for overplus nerve energy. In a future lecture I shall go into this matter in detail, showing you how each muscle of the face, by contracting, produces expressions. A formula in terms of muscular contraction may be easily constructed for hope, fear, rage, hatred, love, joy, etc. Dissimulation does not succeed in masking these expressions always, but it must also be considered. If you care to go deeper into the evolutionary study, it will reveal to you why the sexes appear alike until certain juvenile ages. It will show you that all vertebrate animals are built upon one general plan, and deviate from each other in all particulars by easy gradations. It will not only show you that the "human face divine" is possessed by every mammal at intra-uterine periods, but that man's features are really retrograde affairs.* Man himself has not as good eyes as the falcon, nor has he the swiftness of the deer or olfaction of the hound, though he demonstrably descended from progenitors who were his superiors in these respects.

Darwinism will, it is true, shatter many a false conception upon which the artist doted, but it is merely a substitution of truth for falsehood, fact for glamour. It is not necessary for the artist, to-day, to believe in mythology to enable him to carve Aphrodite or Zeus. Emotion can be as well put upon the canvas—yes, better expressed for being understood, for the painter having recognized the evolved expression through the researches of Gratiolet, Spencer, Huxley, Haeckel and Darwin. The true artistic mind will not shrink from even so repulsive a subject as physiognomy among the insane. Bucknill and Tuke, on Psychological Medicine, afford you a treatise on that head. Instantaneous photography has recently revealed how little our poor eyesight is to be relied upon in determining the true position of animals in motion. Recent periodicals † can be consulted upon this subject, but the artist would blunder sadly if he were to paint animals in motion as the camera sees them, instead of trying to fasten in his picture the impression to which the defective human eye is accustomed. Both Haydon and Flaxman laud the Elgin marbles for their fidelity. There is in them a distinct departure from the legendary methods of chiseling with eyes shut and a master's words ringing in the ears. Better, indeed, to copy nature mechanically, and afterward thoughtfully add "*Il poco piu*" of the finishing hand. "Art is a language. Followed to its legitimate significance, this definition affords at once a test and a suggestion of its character and possibilities; for language is but the medium of ideas, the expression of sentiment—it may be purely imitative or pregnant with individual

* Minot, *American Naturalist*, 1882.

† *Century Magazine*, July, 1882. *Popular Science Monthly*, 1882.

meaning—it may breathe confusion or clearness, emotion or formality, the commonplace or the poetic. The first requisite for its use is to have something to say, and the next to say it well." Durand* asks: "Why should not the American painter in accordance with the principles of self government, boldly originate a high and independent style, based on his own resources?" As to the method thereof, he remarks: "Go first to nature, to learn to paint, and when you have learned to imitate her, you may study the pictures of great artists with benefit. If you ask me to define conventionalism, it is the substitution of an easily expressed falsehood for a difficult truth." Cole wrote on the cover of a sketch-book, soon after his first visit to Europe in 1829:

> "Let not the ostentatious gaud of art,
> That tempts the eye, but touches not the heart,
> Lure me from nature's purer love divine;
> But, like a pilgrim at some holy shrine,
> Bow down to her devotedly, and learn
> In her most sacred features, to discern
> That truth is beauty."

"Who shall presume to imitate the colors of the tulip, or to improve the proportions of the lily of the valley? The criticism which says of sculpture or portraiture that 'Nature is to be exalted, rather than imitated,' is in error. No pictorial or sculptural combination of points of human loveliness do more than approach the living and breathing human beauty as it gladdens our daily path."‡

Byron, who often erred, erred not in saying:

> "I've seen more living beauty, ripe and real,
> Than all the nonsense of their stone ideal."

---

* Tuckerman, Book of Artists, p. 27.

† Op. cit., p. 196.

‡ Edgar Poe, *Landscape Gardener.*

